# Baltimore College of Dental Surgery

**Published:** 1846-03

**Authors:** 


### Miscellaneous Notices.

*Baltimore College of Dental Surgery.*—The annual commencement of this institution for conferring the degree of Doctor of Dental Surgery, was held on the evening of February 17th ult. in the presence of a crowded audience.

The exercises were opened with prayer by the Rev. Dr. Dorsey, after which the band played a tune, when commenced the interesting ceremony of conferring the degree, which was done by the Dean of the Faculty, calling up in alphabetical order the several students who were to receive this honor—when after announcing the states and counties of each one's residence, together with the subject matter of their several theses, the authority for conferring the degree was then read, after which the diploma of the college was presented to each by Dr. John Harris.

Music again enlivening the scene—Prof. C. A. Harris then rose and addressed the graduates in reference to the new theatre of action upon which they were about to embark, and warned them of the responsibilities, duties, and obligations which would constantly claim their attention.

After the address, Mr. Clarkson, one of the graduating class, arose, and in behalf of his fellow graduates and the dental class, returned their thanks to the Faculty for their unremitted efforts to instruct them in the several branches of dental science, together with the kindness and attention uniformly extended to them.

There were nine received the degree—the largest number since the foundation of the college, and it may not be uninteresting to state here the names of the several graduates, with their residence and theses. They are as follows, viz.

Wilkes Allen, Massachusetts; Diseases of the Maxillary Sinus.
Stephen Parsons, Georgia; on the Extraction of Teeth.
E. P. Burroughs, Canada; Morbid Effects of Diseased Teeth.
R. W. Clarkson, N. Jersey; Healthy and Morbid Sympathy.
Wm. F. Bason, N. Carolina; Preservation of the Temporary Teeth.
A. Baldwin, M. D., Alabama; Constitutional Effects of Diseased Teeth.
J. W. Neil, London; Morbid Effects of Diseased Teeth and Gums.
V. M. Swaze, N. Jersey; on the Extraction of Teeth.
John Locke, Pennsylvania; on Filling Teeth.

